# Extensively hydrolyzed versus intact milk protein formula and risk of necrotizing enterocolitis: a meta-analysis

**DOI:** 10.3389/fped.2026.1849871

**Published:** 2026-06-03

**Authors:** Puja Kulkarni, Sarah Tang, Amy Chan, Riley Danna, Jose Puglisi, Yinka Davies

**Affiliations:** California Northstate University College of Medicine, Elk Grove, CA, United States

**Keywords:** amino acid formula, feeding intolerance, formula, hydrolyzed formula, necrotizing entercolitis, preterm infant, standard intact protein formula

## Abstract

**Background:**

Necrotizing enterocolitis is a life-threatening inflammatory condition that commonly affects the intestinal wall of neonates. Feeding intolerance, rectal bleeding, and bowel perforations are predominant findings of the pathology, of which the etiology is not fully understood. Multiple factors are speculated to contribute to this disease, including formula feeding. Nutrition plays a critical role in neonatal outcomes, and formula composition significantly influences gastrointestinal health and development. This meta-analysis evaluated the impact of extensively hydrolyzed formulas (eHFs) compared to standard intact protein formulas (SPFs) on the incidence of necrotizing enterocolitis (NEC) and feeding intolerance (FI) in preterm infants.

**Methods:**

A comprehensive literature search was conducted across Ovid, Cochrane and PubMed: the search terms included combinations of “Necrotizing Enterocolitis,” “Hydrolyzed,” “Amino Acid,” “Intact,” “Standard,” and “Formula” alongside preterm-related keywords. Eligible studies included randomized controlled trials comparing eHF and SPF diets in preterm infants, defined as being born at less than 37 weeks gestation. Primary outcomes were the incidence of NEC and FI. Odds ratios (OR) for dichotomous outcomes were calculated using a chi-square analysis with 95% confidence intervals (CIs).

**Results:**

Three randomized controlled trials, consisting of a total of 1,180 preterm infants, met the inclusion criteria for this study. Infants that received SPFs had a statistically significant increased risk of NEC (OR=2.54; 95% CI = 1.38–4.68) and FI (OR=1.87; 95% CI = 1.38–2.52) compared to those fed eHFs. Sensitivity analyses confirmed the robustness of these findings.

**Conclusion:**

Standard intact protein formulas significantly increase the risk of NEC and FI in preterm infants compared to extensively hydrolyzed formulas. These findings support the incorporation of eHFs into neonatal feeding guidelines. Previous studies have also highlighted the role of partially hydrolyzed whey formula and extensively hydrolyzed cow’s milk protein formula in preventing allergic pathologies, such as atopic eczema, in children up to age 10 without rebound, indicating that the effects of formula extend well beyond infancy. While further research is needed to evaluate long-term clinical outcomes, this research calls for the critical need to refine infant formula manufacturing and feeding guidelines.

**Systematic Review Registration:**

https://www.crd.york.ac.uk/PROSPERO/view/CRD42025641145.

## Introduction

Necrotizing enterocolitis (NEC) is a life-threatening inflammatory condition that commonly affects the intestinal wall of neonates (1). The disease involves inflammation, necrosis, and eventual disintegration of the intestinal lining (2). Feeding intolerance (FI), rectal bleeding, and bowel perforations are predominant findings of the pathology (3, 4). NEC affects roughly 5% of all premature infants and accounts for 8% of neonatal intensive care unit (NICU) admissions in the United States, with a mortality rate reaching up to 50% (5). Despite its severity, the exact etiology remains unclear; identified risk factors include prematurity, low birth weight, congenital defects, and formula feeding (6, 47, 48).

Infant nutrition plays a critical role in neonatal outcomes, and formula composition significantly influences gastrointestinal health and development. Available formula types include standard intact protein formulas (SPFs), partially hydrolyzed formulas (pHFs), extensively hydrolyzed formulas (eHFs), and amino acid-based formulas (AAs) (7). SPFs, which are cow's milk based, contain lactose, vegetable oils, minerals, vitamins, and a whey:casein ratio of 60:40 (8). In eHFs, proteins are broken down into smaller peptides (<3,000 Daltons) through processes such as enzymatic hydrolysis, ultrafiltration, and heat-treatment (9). If the proteins are only partially broken down, pHFs are produced (10). In contrast, if the proteins are extensively hydrolyzed into their individual amino acid components, AAs are created (11). Although formulations differ across brands, the degree of protein breakdown is a consistent distinguishing feature (12). Hydrolyzed formulas are considered safe and well-tolerated with mild transient side effects such as increased stool frequency and more green-colored stools, likely as a result of the smaller protein size. There has been concern that eHF may slow weight gain in the first year of life, but multiple studies have shown that hydrolyzed formulas allow for adequate growth comparable to SPF and breast-fed infants (13, 14).

Current formula feeding guidelines in NICUs tend to utilize SPFs for enteral nutrition and supplementation (12). This is also reflected in federally funded programs such as WIC, the USDA’s special supplemental nutrition program for Women, Infants, and Children (15). In California, all formulas offered through WIC to low income and nutritionally at risk populations are SPFs (16). Despite this uniformity in practice, there is little research comparing the effects of different formula compositions on clinical outcomes, particularly in conditions with high morbidity and mortality, such as NEC.

To address this, this analysis aimed to evaluate the impact of eHFs compared to SPFs on the incidence of NEC and FI in preterm infants.

## Methods

The study protocol was registered on PROSPERO on 02/05/2025 (CRD42025641145). The study was conducted and reported according to the guidelines established by Preferred Reporting Items for Systematic Reviews and Meta-analyses (PRISMA) (17).

### Search strategy and study selection

We conducted a search across PubMed, Cochrane, and Ovid from inception to 01/27/2025 without restrictions. Search terms included the types of cow's milk protein formula (extensively/partially hydrolyzed, amino acid, or standard/intact), necrotizing enterocolitis, and preterm infants/neonates.

Eligible studies consisted of randomized controlled trials and cohort studies that compared an intervention group receiving a form of hydrolyzed cow's milk protein formula (eHF, pHF, or AA) to a control group receiving SPF in preterm infants, defined as less than 37 weeks gestational age, with NEC as a reported outcome. Studies examining full-term infants and/or formulas not derived from cow's milk protein were excluded.

### Data extraction

Search results were uploaded to Covidence, a tool used to conduct coordinated screening and data extraction for systematic reviews. Four reviewers independently conducted title and abstract screening, with each article reviewed by two reviewers. Conflicts were resolved by a third reviewer. Next, a full-text review and eligibility assessment followed the same protocol. From the eligible studies, data was extracted onto a table that included spaces for the study title, authors, publication year, sponsors, study design, country, population studied, intervention and control groups, and various neonatal outcomes.

### Bias assessment

Randomized controlled trials were evaluated using the Revised Cochrane risk-of-bias tool for randomized trials (RoB 2), published in 2019 by Higgins et al. (18) Cohort studies were evaluated using the Risk Of Bias In Non-randomized Studies of Interventions, Version 2 (ROBINS-I V2) tool, published in 2024 by Sterne et al. (19) Two authors independently assessed each article for risk of bias. Randomized controlled trials were evaluated in the following domains: randomization process, deviations from intended interventions, completeness of outcome data, measurement of outcome, and selection of the reported result. Besides the relevant domains mentioned, cohort studies were also evaluated for: confounding variables, classification of interventions, and participant selection. Disagreements were resolved by discussion amongst the review team. The bias assessment results are summarized in Tables 1, 2.

**Table 1:** Risk of bias analysis for included RCTs.

Paper	Risk of bias domain					Overall risk of bias
	Randomization process	Deviations from intended interventions	Missing outcome data	Measurement of outcome	Selection of reported result	
Li et al., (20)	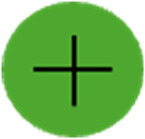	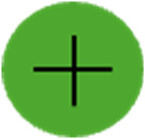	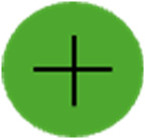	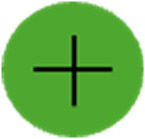	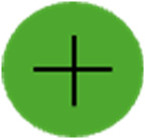	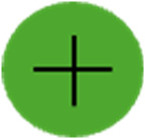
Yin et al., (21)	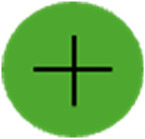	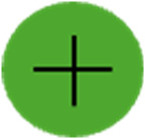	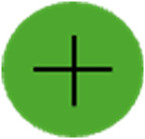	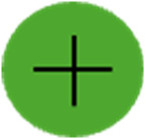	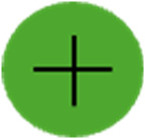	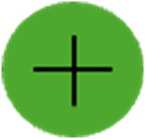

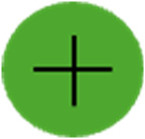
 Low Risk.

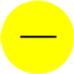
 Some Concerns.

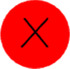
 High Risk.

**Table 2:** Risk of bias analysis for included cohort study.

Paper	Risk of bias domain							Overall risk of bias
	Confounding	Classification of interventions	Selection of participants	Deviations from intended interventions	Missing data	Measurement of outcome	Selection of reported result	
He et al., (9)	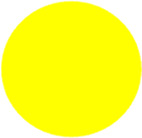	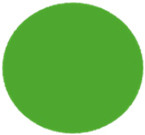	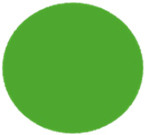	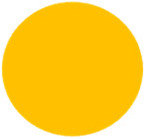	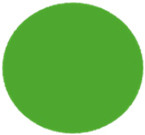	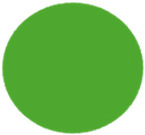	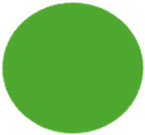	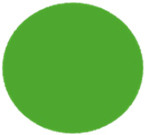

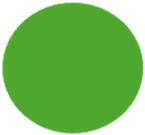
 Low risk of bias.

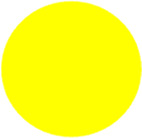
 Low risk of bias except for concerns about uncontrolled confounding.

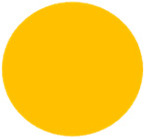
 Moderate risk of bias.

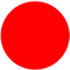
 Serious risk of bias.

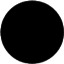
 Critical risk of bias.

### Data synthesis and analysis

The primary outcomes were the incidence of NEC and FI in infants fed SPFs compared to those fed eHFs.

From each of the three eligible studies, we extracted the number of infants who developed NEC and FI in both the SPF and eHF groups, as well as the total number of infants in each group. All infants included were less than 37 weeks gestational age, with the average age across studies being 31 weeks gestation. Using these data, we constructed 2 × 2 contingency tables for each study and calculated odds ratios (ORs) with 95% confidence intervals (CIs) to estimate the relative odds of NEC or FI associated with SPF versus eHF feeding.

A random-effects meta-analysis model was used to pool the ORs across studies, accounting for potential between-study variability. Statistical heterogeneity was assessed using Cochran's *Q* test, the I^2^ statistic, and the between-study variance (*τ*^2^). An I^2^ value greater than 50% was considered indicative of substantial heterogeneity.

## Results

### Study selection

**Figure 1:**
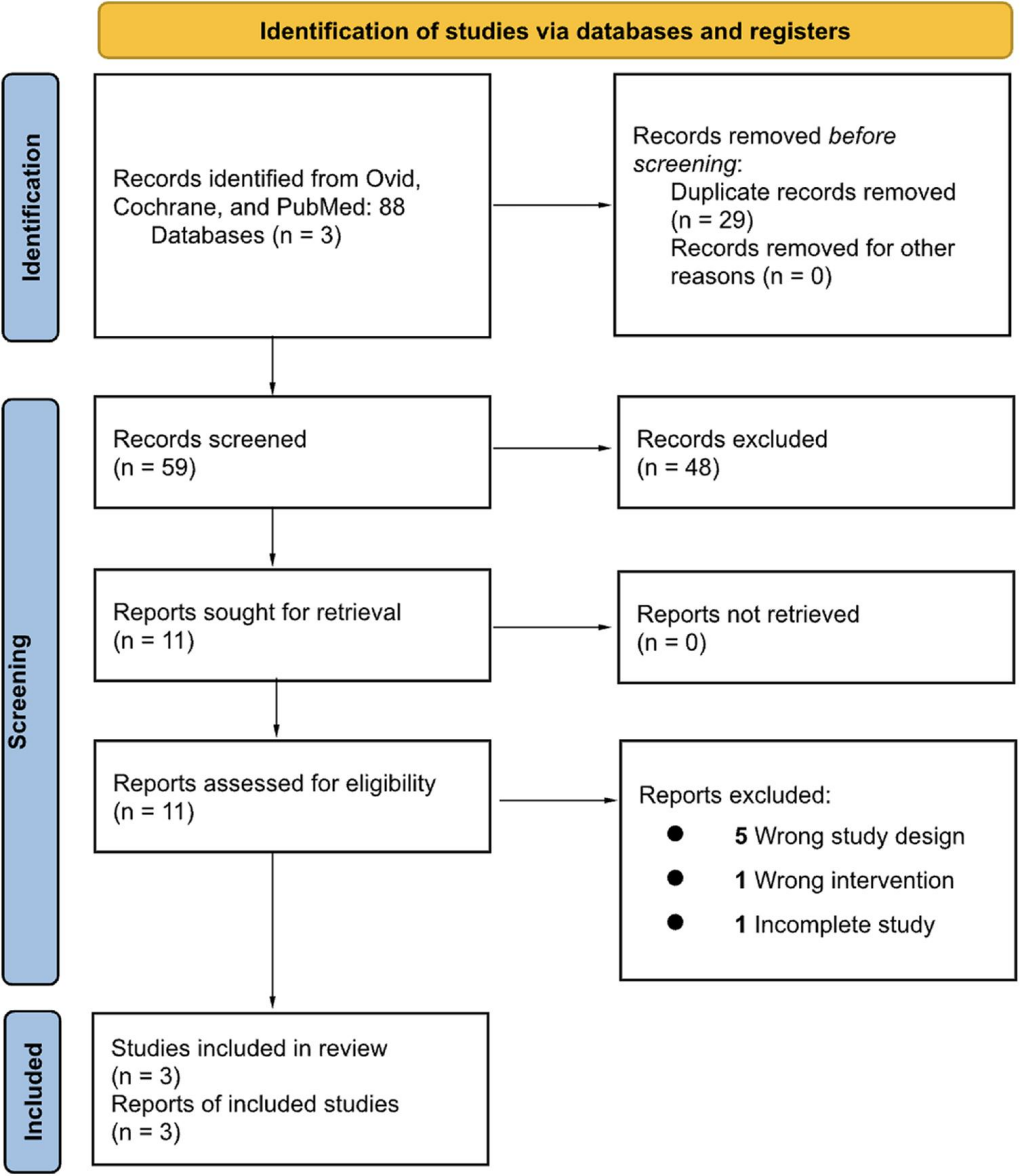
Flow diagram of study selection.

The initial study yielded 88 records from Ovid, Cochrane, and PubMed databases. After removal of 29 duplicates, 59 records underwent title and abstract screening. Of these, 48 records were excluded due to lack of NEC data or inappropriate study design. Ultimately, three studies were included in the meta-analysis (Figure 1) (9, 20, 21).

### Study characteristics

Among the three studies included in the analysis, a total of 1,180 preterm infants were assessed.

Two of the studies were blinded randomized controlled trials, while the third was a retrospective cohort study. Despite differences in study design, the populations, interventions, and outcome measures were sufficiently similar to permit combined analysis.

### Risk of bias

All included studies were conducted in a single country, which may limit the generalizability of the findings to the global population. Risk of bias was judged to be low to moderate in the studies based on study design and outcome reporting — including randomized controlled trials and retrospective studies. One additional study (Baldassarre et al.) was excluded due to high risk of bias related to industry sponsorship, as the trial was funded by a formula manufacturer and lacked outcome assessor independence (22). The results of the bias analysis on the three included studies are summarized in Tables 1, 2.

### Analysis results

**Figure 2:**
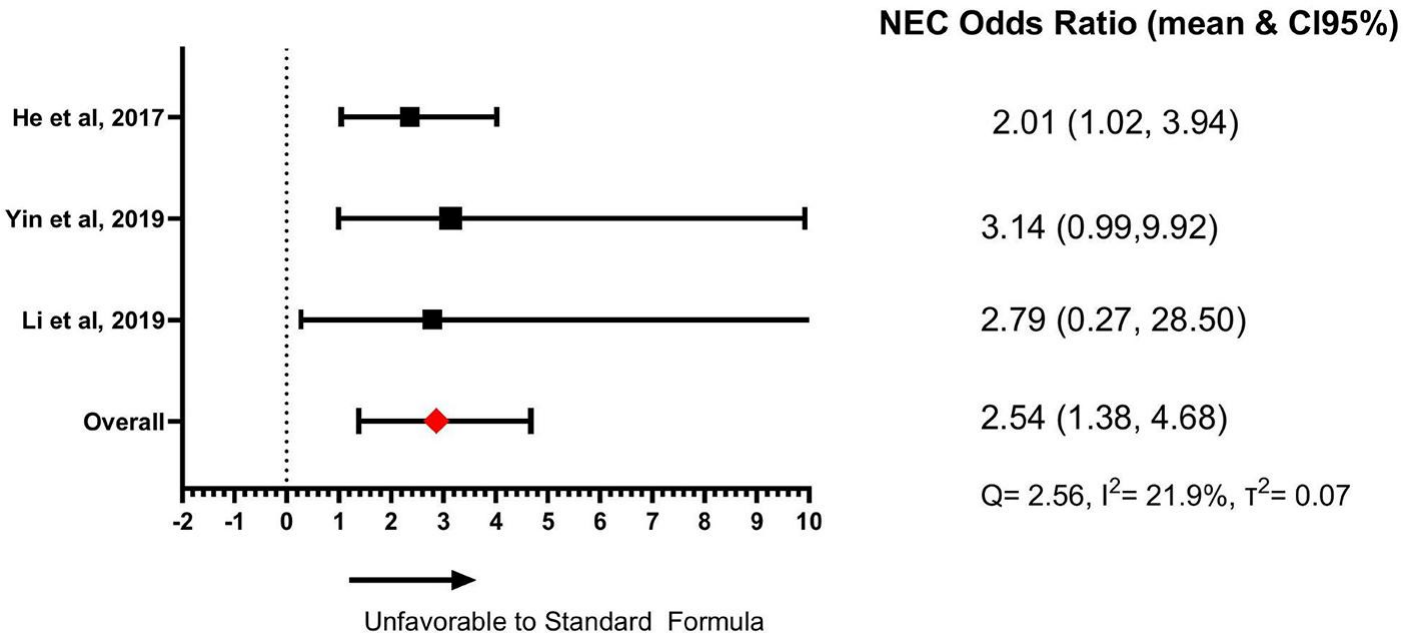
Forest plot of odds ratios for NEC in preterm infants fed SPFs versus eHFs across three studies.

**Figure 3:**
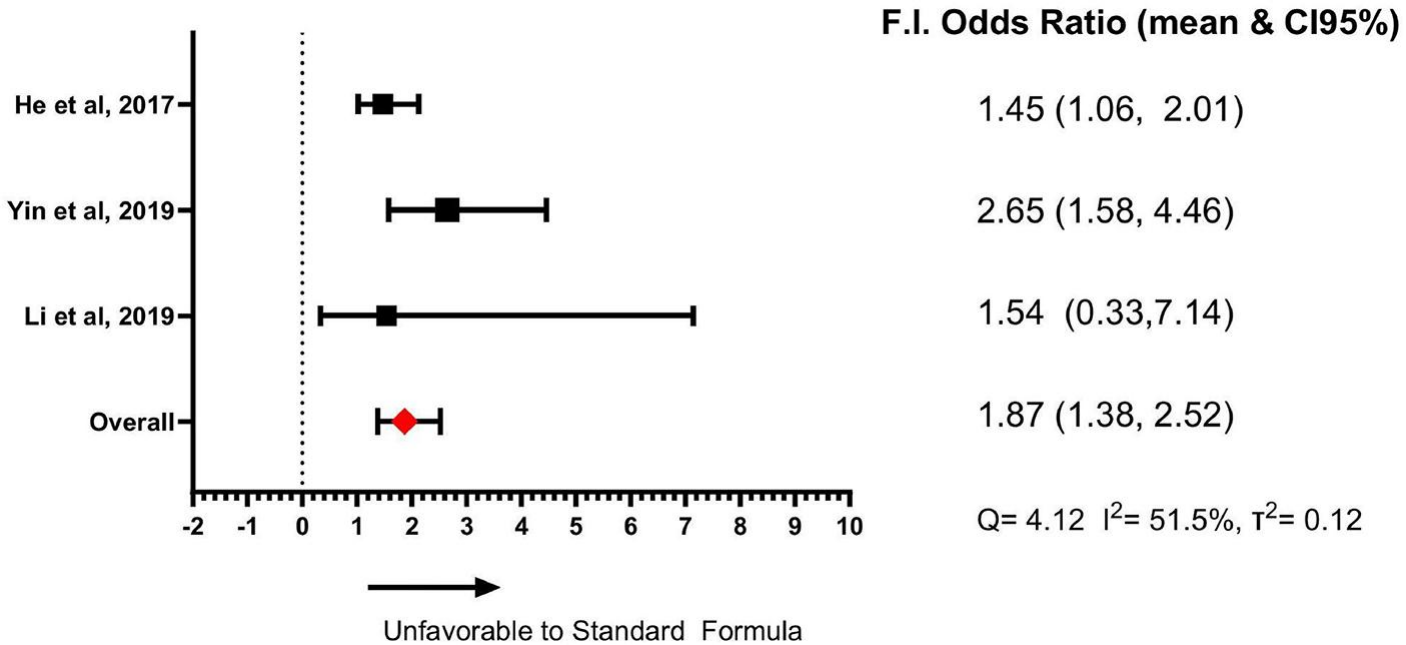
Forest plot of odds ratios for FI in preterm infants fed SPFs versus eHFs across three studies.

Meta-analysis revealed a significantly increased risk of NEC and FI among infants fed SPFs compared to those fed eHFs. As depicted in Figure 2, the pooled odds ratio for NEC was 2.54 (95% CI: 1.58–4.68), indicating that infants fed SPFs were more than twice as likely to develop NEC compared to those receiving eHFs. Some of the studies reported an OR as high as 3.14 (21) and the minimum value was 2.01 (9), with a 21% heterogeneity (I^2^) overall. Figure 3 shows the overall odds ratio for FI with a mean value of 1.87 (95% CI: 1.38–2.52), indicating an 87% higher risk of developing FI with SPFs. Individual study estimates analyzing the risk of FI ranged from an OR of 1.45 (9) to 2.65 (21), with one study showing a wider confidence interval and less precision due to smaller sample size (20). Heterogeneity was moderate (I^2^ = 51.5%).

### Summary of findings

The combined results from all three studies consistently showed that SPFs were associated with significantly higher risks of both NEC and FI in preterm infants. Sensitivity analyses confirmed the robustness of these findings, indicating consistent associations between SPFs and increased risks of both FI and NEC.

## Discussion

This study investigated how formula type influences the risk of NEC and FI in preterm infants, specifically comparing eHFs to SPFs. Infants fed SPFs demonstrated a significantly higher incidence of both NEC and FI than those fed eHFs. Although the underlying mechanisms remain unclear, several characteristics of eHFs may contribute to their protective effects, including reduced antigenicity, enhanced gastric emptying (23), improved gastric motility, and easier digestion due to its smaller molecular weight (24–26). Further research into the physiologic mechanisms of various formulas is needed to elucidate the protective phenomenon exhibited by eHFs; nonetheless, these results support the incorporation of eHFs into neonatal nutritional protocols as a potential strategy to reduce the risk of gastrointestinal complications in preterm populations. Future studies are required to account for factors such as gestational age, birth history, and family history into individualized nutrition plans for preterm infants.

The significance of our findings is especially pertinent in light of current formula feeding guidelines for preterm infants, with most protocols utilizing SPFs (12). Government-supported nutrition programs also mostly provide SPFs, including WIC (15). This may be due, in part, to the higher cost associated with hydrolyzed formulas (27). For example, Enfamil AA formula, which is marketed for babies with severe food allergies, is priced at $0.63 per fluid ounce, compared to $0.27 per fluid ounce for the brand's SPF (28). This price difference could indicate a higher production cost for companies, but no clear manufacturing analyses have been released to the public to support this (29). The cost of treating NEC must also be brought into consideration. Based on a retrospective analysis from 2009 to 2018 of children's hospitals in the Pediatric Health Information System, the median cost of treating surgical NEC was $430,860 per infant, ranking as one of the costliest morbidities associated with prematurity (30). When looking at the long-term healthcare costs for infants enrolled in Texas Medicaid from 2002 to 2003, the cost of treating NEC medically was $5112 higher per infant than matched controls between 6 and 12 months of age. The mean incremental cost for infants with NEC treated surgically was even greater at $18,274 from age 6–12 months. For these infants, healthcare costs remained higher than for matched controls through 36 months of age (31). Thus, using the less expensive formula option may not ultimately be the most cost efficient economically. Formal cost-benefit analyses should be conducted to assess the feasibility of implementing eHFs across diverse healthcare systems in order to support this potential advantage.

Along with the severity of early life pathologies, such as NEC, formula hydrolyzation has been associated with long-term benefits that extend beyond infancy into childhood, including growth trajectories, neurodevelopmental outcomes, and various other pediatric pathologies (10, 24, 32–38). The German Infant Nutritional Intervention (GINI) study investigated the role of hydrolyzed formulas in preventing allergic diseases among exclusively formula-fed infants with a family history of atopy (39, 40). The study found that the incidence of atopic dermatitis was significantly reduced in children fed eHFs compared to SPFs, with this protective effect persisting up to 10 years of age without rebound (40). Additionally, the degree of hydrolyzation played a role in the outcomes: while pHFs also conferred benefit, their effect was less pronounced than that of eHFs (40). It was also observed that some children experienced a protective effect against the development of asthma and allergic rhinitis with the use of eHFs, though these findings were less conclusive (40). These pathologies are driven by eosinophils (41), and in Li et al., infants fed eHFs had significantly lower eosinophil percentages before discharge compared to the SPF group (20), further reinforcing the link between formula type and immune modulation. Although allergic conditions do not carry the high mortality risk associated with NEC, the GINI study forms the foundational basis for this meta-analysis, providing further evidence that SPFs are linked to negative childhood outcomes when compared to hydrolyzed formulas.

The applicability of our findings may be constrained by the limited number of studies included in this meta-analysis. A general scarcity of research on this topic made it challenging to find additional data that met all inclusion criteria. Consequently, all studies analyzed were in a single country, which restricts the diversity and generalizability of the results. Additionally, all three studies exclusively examined eHFs, despite the availability of formulas with varying degrees of hydrolysis on the market–including pHFs and AA-based formulas. Future studies should incorporate these additional formula types to better reflect clinical diversity and assess their comparative effectiveness (9, 20, 21). Due to the small number of eligible studies incorporated in our meta-analysis, a flexible inclusion criteria was adopted. For example, He et al. conducted a retrospective cohort study that defined the eHF group as infants who were initially fed 150 mL/kg of eHF, then switched to the same volume of SPF for continued feeding (9). This protocol introduces a slight deviation from intended interventions and may confound the analysis, as the eHF group ultimately received both formula types.

Each of the papers included in our analysis observed the incidence of feeding intolerance; however, the definitions of feeding intolerance varied between each paper. In the U.S., feeding intolerance is defined as the presence of at least one of the following symptoms: severe abdominal distention, signs of intestinal perforation, hematochezia, gastric retention, bile reflux or vomiting, bradycardia, and severe cardiopulmonary insufficiency (42). Two of the papers adhered to this definition (20, 21), while one paper required the presence of at least two symptoms for diagnosis (9), potentially compromising the comparability of the studies’ outcomes. Since FI is diagnosed clinically and inherently subjective, often dependent on physician interpretation, these definitional discrepancies should be acknowledged, though they are unlikely to undermine the broader conclusions.

Another limitation is the lack of consistency in formula specifications and feeding regimens. Only one study explicitly stated the formula brands used: Nestle Ales (caloric density 71 kcal/100 mL, protein content 2.1 g/100 mL) and Similac Special Care (caloric density 68 kcal/100 mL, protein content 2.1 g/100 mL) (20). The other two studies did not specify brands, making it difficult to account for potential formulation differences (9, 21). Similarly, the exact timeline of feedings (initiation, time between feedings, etc.) differed between papers. While the general distinction between eHF and SPF remains valid, future research should explicitly report product details, including brand, nutritional content, and feeding regimens.

A further concern is potential bias introduced by industry sponsorship. During the review process, several studies in this field were identified as having funding by formula companies. One such study, sponsored by Mead Johnson Nutrition, a company primarily selling SPFs, reported no significant difference in NEC incidence between eHF and SPF but was excluded from our analysis due to concerns of a conflict of interest (22, 43). Similarly, a 2019 systematic review investigating the topic of our study concluded that the type of formula had no difference on the risk of NEC (27). However, the papers analyzed in this review, including the sponsored paper referenced above, were not devoid of investment by formula corporations, so we determined there is still value in reexamining this research question. This highlights the commercial influence in infant nutrition research. Given that four companies control 90% of U.S. formula manufacturing, valued at $3.96 billion in 2022, research in this field may be underreported or influenced by market interests (44, 45). Subsequent investigations into this topic, including systematic reviews, should explicitly report funding sources and evaluate their potential effects on relevant conclusions.

Amid current feeding protocols and limited unbiased literature, there is a pressing need to expand research into the effects of formula composition on both neonatal and long-term health outcomes. This urgency is underscored by ongoing lawsuits alleging that cow's milk-based formulas have contributed to NEC-related injuries and deaths in preterm infants (46). The need for a consensus in this area of research is becoming more pertinent as the formula chosen by parents and providers shows a propensity to affect the health of a newborn well into adulthood.

## Conclusion

SPFs significantly increase the risk of NEC and FI in preterm infants compared to eHFs. These findings support the incorporation of eHFs into neonatal feeding guidelines. While short-term outcomes favor the use of eHFs, further research is needed to evaluate long-term clinical and developmental outcomes, including growth trajectories, nutrient absorption, and neurodevelopment. Additionally, studies should explore the cost-effectiveness and accessibility of eHFs, as well as their role in individualized nutrition plans that account for gestational age, birth weight, and comorbidities. To improve outcomes and inform evidence-based clinical practice, larger, multi-center studies are needed to validate existing data and ensure generalizability across diverse neonatal populations. Refining feeding strategies through ongoing evidence-based research is essential to optimizing care for preterm populations.

## Data Availability

The original contributions presented in the study are included in the article/Supplementary Material, further inquiries can be directed to the corresponding author.
